# Practice of Comparative Effectiveness Research to Identify Treatment Characteristics of Similar Chinese Patent Medicine for Angina Pectoris

**DOI:** 10.1155/2017/7062714

**Published:** 2017-08-13

**Authors:** Hongbo Cao, Ping Wang, Nan Li, Dan Liu, Junshao Ma, Ruihong Fan, Zhihuan Zhou

**Affiliations:** ^1^Tianjin University of Traditional Chinese Medicine, Tianjin, China; ^2^Center for Evidence-Based Medicine, Tianjin, China; ^3^Wuqing Hospital of Traditional Chinese Medicine, Tianjin, China; ^4^Nakai Hospital, Tianjin, China; ^5^The Hospital of Traditional Chinese Medicine, Tianjin, China

## Abstract

**Objective:**

Individualized application of TCM is not easy and may lead to undesirable results, such as poor effect or even adverse reactions. This trial aims to compare two common Chinese patent medicines with similar effects.

**Background of the Research:**

Four hospitals carried out the test at the same time in Tianjin city of China.

**Participants:**

144 patients were involved in this study; all patients must meet the diagnostic criteria.

**Interventions:**

Qishen Yiqi pills, compound danshen pills, and their placebos; an efficacy analysis was conducted after the first medication and after crossover medication.

**Primary Outcome Measures:**

The primary index of end point includes Seattle Angina Questionnaire score-7 and score of 7-point Likert Scale; the curative effect was compared with minimal clinically important differences value.

**Result:**

Two drugs have their respective advantages in treating SAP. In practical application, the two drugs shall be discriminated in use based on patients' specific symptoms.

**Trial Registration:**

Chinese clinical trials register is ChiCTR-TTRCC-14004406 (registered 23 March 2014).

## 1. Background

CHD (Coronary Heart Disease) and AP (Angina Pectoris) are global public health problems, posing a big threat to the life quality and safety [1] of humanity, and their incidence is going up [2] year by year. Practices indicate that Traditional Chinese medicines boast distinctive advantages [3] in treating CHD and AP or in delaying its deterioration. They are most notable in relieving or improving clinical symptoms [4] of patients, avoiding drug resistance, reducing adverse reactions, and shortening days of hospitalization; hence they are more desirable for long-term use [5]. Owning to these advantages, they have won favor and recognition of many patients and have found wide application in China [6].

Randomized Controlled Trial (RCT), a prevailing standard for comparing clinical intervention effect in the world, is often used for comparing the efficacy of two drugs. However, the evidence thus acquired is the mean clinical value based on a specific population, and some may not be appropriate for deciding individual treatment in clinical practices. Even subgroup analysis is also very difficult to solve the problem [7]. Nevertheless, drug personality and individualized therapy represent the trend of international medicine [8].

Traditional Chinese medicine is a clinical medicine evolved from individualized diagnosis and treatment [9]. Treatment based on syndrome differentiation, the core of TCM clinical treatment [10, 11], features a general analysis of individual patients and then prescription of different therapies. For a long time, to adapt itself to modern preparation technology, a large amount of Chinese patent medicines have been developed, pushing the number of medicines for curing the same disease up to several dozens or even a hundred [12]. For instance, there are as many as 349 similar drugs for treating CHD and AP [13]. However, the differentiation of efficacies across varieties is not clearly defined. The development of a large variety of Chinese patent medicines is meant to meet the demands of TCM for syndrome differentiation and facilitate the use by individuals, but 70% of Chinese patent medicines are used by non-TCM persons (western medicine staff and patients) [14, 15]. A lack of TCM knowledge has caused confusion in the clinical use of Chinese patent medicines, poor efficacy, and even adverse reactions.

CUPID is a novel evaluation method built in such a context by our team [16]. Taking therapeutic effect on symptoms as a breakthrough and based on traditional RCT and semicross design, this method conducts CER (Comparative Effectiveness Research) of two Chinese patent medicines from different treatment levels by setting PIO (Patient Important Outcome) and PRO (Patient Report outcome) of patients [17, 18]. In the end, it provides a general description of the individual curative characteristics of different drugs, the easiest to understand, making it easier for non-TCM persons to select and use the drugs.

Compound danshen pills and Qishen Yiqi pills, two common Chinese patent medicines for treating CHD and AP, are known for their wide clinical use and stable efficacy. This trial aims to compare the clinical advantages and characteristics of the two drugs based on the two most common TCM syndromes of CHD and AP, known as “Qi deficiency and blood stasis” and “Qi stagnation and blood stasis.”

## 2. Trial Design

This trial is designed to be a randomized controlled, double-blind double dummy, and semicrossover trial.

### 2.1. Screening of Participants

**Figure 1:**
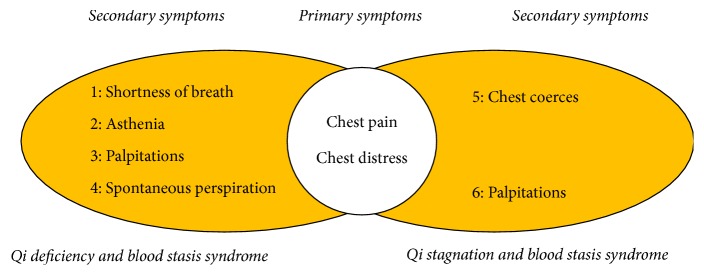
Syndrome and symptomatic combination types.

Participants must meet the AP diagnostic criteria of ischaemic heart disease (refer to World Health Organization diagnostic criteria for International Hydrological Decade and the classification criteria of Canada on International Hydrological Decade) and fall into “Qi deficiency and blood stasis” and “Qi stagnation and blood stasis” syndromes (refer to Guiding Principles for Clinical Study of New Chinese Medicines 2002); patients must be aged between 40 and 75 and must have signed informed consent; the excluded patients are those who have attended or are attending other clinical trials in recent 3 months, those of allergic constitution, those allergic to some known contents of said drugs, those considered inappropriate by researchers, those complicated with active peptic ulcer and other hemorrhagic disease, pregnant women or women preparing for pregnancy and lactating women, those complicated with Grade III hypertension and serious arrhythmia uncontrolled, those complicated with severe primary diseases with hepatic, renal, and hematopoietic system and mental disease and malicious tumor, and those identified with serious mistakes or errors, voluntary withdrawers, and those with serious safety accidents. The trial shall be terminated at once in case of serious adverse events (AEs) or major mistakes with the plan (Figure 1).

### 2.2. Randomized Blinding

A third-party statistician prepared the randomized allocation table using SAS 9.1 statistic software; patients were allocated (1 : 1) at random using stratification and random block design; stratification factors are syndrome type (“Qi stagnation” and “blood stasis and Qi deficiency and blood stasis”) and symptomatic assemblage type (primary symptoms + secondary symptoms, primary symptoms are chest pain and chest distress and secondary symptoms are chosen by patients themselves; they are divided into 6 groups in all by 2002 Guiding Principles for Clinical Study of New Chinese Medicines; thus, 17 subgroups are divided in all); drugs were blinded according to the random number table, by persons not involved in the trial; the blind codes were made into two copies to be kept by major study unit and the applicant unit, respectively.

### 2.3. Sample Size

The sample size was calculated on the basis of literature research. The means of the Seattle angina questionnaire (SAQ) scores was assumed to be the group participants. Subjects Taking Qishen Yiqi pills and taking compound danshen pills were 82 and 80, respectively, with the same SD (SD = 4). To show a significant intergroup difference, two groups of patients totaling 144, 72 in each group should be included, adopting significance level of 0.05, 80% power and a drop-out rate of 15% using the PASS software.

### 2.4. Grouping Method

From December 2014 to June 2015, the study team recruited 144 AP patients from 4 hospitals of Tianjin (Tianjin hospital of TCM, Baokang Hospital of Tianjin University of TCM, Tianjin Nankai Hospital, and Wuqing hospital of TCM in China) willing to join in the trial and meeting the inclusion criteria (72 patients falling into “Qi stagnation and blood stasis” syndrome, 12 patients in each symptomatic assemblage type; 72 patients falling into Qi deficiency and blood stasis syndrome, 12 members in each symptomatic assemblage type). Basic data of patients was archived, and patients were grouped at one time within 3 days after the trial commenced.

### 2.5. Method of Intervention

#### 2.5.1. Intervention Drugs

Drug A is Qishen Yiqi pills + compound danshen pills as placebo; drug B is compound danshen pills + Qishen Yiqi pills as placebo; Drug C is Qishen Yiqi pills as placebo + compound danshen pills as placebo (test drugs and simulation drugs are both provided by Tasly Pharmaceutical Group Co., Ltd; the two are basically identical in appearance, shape, and color).

#### 2.5.2. Intervention Phase

First medication lasted 14 days, 3-day washout period (according to references, the major components of compound danshen pills and Qishen Yiqi pills can be completely metabolized in human bodies within 3 days) [19–21] and 14-day crossover medication.

All patients are on normal use of regular medicines; first efficacy analysis was conducted after the first medication. The effective population terminated medication, and the ineffective population passed the washout period and entered crossover medication observation; a comprehensive analysis was made after the crossover medication.

#### 2.5.3. During the Trial Period

During the trial period, Participants were not allowed to take any other Chinese patent medicines or herbal medicines with similar effect of activating blood circulation and removing blood stasis.

### 2.6. Index of End Point

Score of SAQ (Seattle angina questionnaire score)-7 and Score of LS (Score of 7-point Likert scale) [18] are given.

### 2.7. Follow-Up Visit

All patients were required to be followed up at 0 day, 14 + 1 day, 17 + 1 day, and 31 + 1 day after entering the group.

### 2.8. Outcome Measure

#### 2.8.1. SAQ Method

(1) SAQ=latitude  1  score+latitude  2  score+latitude  3  score.

Latitude 1 score = 100*∗*(total  score  of  the  three  items − 3)/15; latitude 2 score (item 3, 4) = 100*∗*(total  score  of  the  two  items − 2)/10; latitude  3  score  (item  9, 10) = 100*∗*(total  score  of  the  two  items − 2)/8.

#### 2.8.2. MCID (Minimal Clinically Important Differences) Method

Select patients whose Likert scale scored between +2 and +3, extract relevant SAQ score and define it as variable *X*_1_, calculate the mean value ${\overset{-}{X}}_{1}$, choose patients whose Likert scale scored between 0 and +1, extract relevant SAQ score and define it as variable as *X*_0_, and calculate the mean value ${\overset{-}{X}}_{0}$ (see (2)).

(2) $$\begin{array}{c} MCID=\sqrt{\frac{{\sum \left(X_{1}-{\overset{-}{X}}_{1}\right)}^{2}+{\sum \left(X_{0}-{\overset{-}{X}}_{0}\right)}^{2}}{\left(n-1\right)}}\times \left(\;1-r\;\right), \end{array}$$

where *n* represents the sample size and *r* represents the reliability coefficient of SAQ scale.

#### 2.8.3. Outcome Measuring Method

Compute the total SAQ score of each patient at the baseline and at the end of phase I, mark them as SAQ1 and SAQ2: If SAQ2 − SAQ1 > MCID, it is decided as effective. If SAQ2 − SAQ1 < MCID, it is decided as ineffective.

### 2.9. Statistical Analysis

This study adopts spss 16.0 statistical analysis software for statistics analysis and chi-square test for intergroup comparison of qualitative materials and adopts t test for intergroup comparison of quantitative materials and uses CA (Correspondence analysis) method to screen out the optimal clinical symptoms of drugs.

## 3. Results of the Study

A total of 144 cases were included and 119 effective cases entered the final statistical analysis. Among all, 8 persons were excluded for not conforming to the inclusion criteria, 10 persons withdrew the test for poor efficacy, 4 persons were excluded for violating the plan, and 3 persons dropped out for adverse events.

### 3.1. Intergroup Comparison of Baseline Population Characteristics

See Table 1.

### 3.2. Individual Evaluation of Outcome

**Figure 2:**
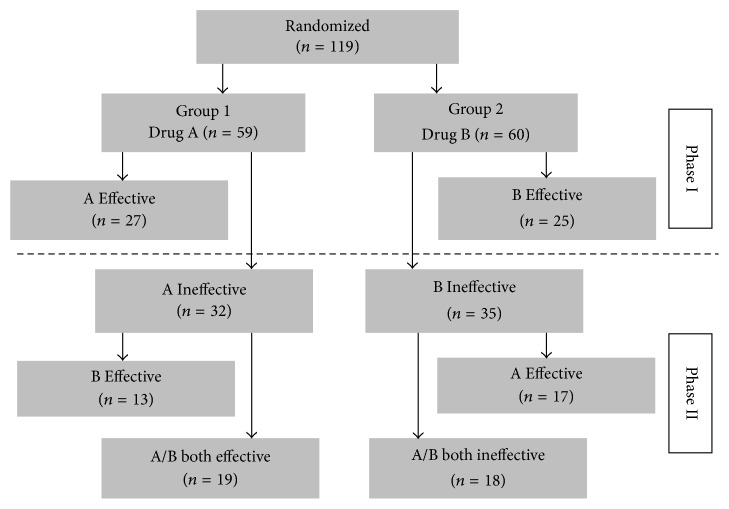
Individual evaluation of outcome.

From the above formula, MCID value we obtained is 2.0. With MCID value as the cut-point, we measured the efficacy of the drugs. The results indicate (Figure 2), in the first phase, that the drug was found effective in 27 persons and ineffective in 32 persons in group 1 (drug A); the drug was effective in 25 persons but ineffective in 35 persons in group 2 (drug B). A total of 52 patients discontinued the medication and 67 patients needed to continue the treatment after changing medication; in the second phase, the drug was effective in 13 persons and ineffective in 19 persons in group 1 (drug B), and the drug was effective in 17 persons and infective in 18 persons in group 2 (drug A).

### 3.3. Subgroup Analysis

Consolidated classified outcomes by different syndromes, symptoms/syndrome complex for subgroup analysis (Table 2).

### 3.4. Correspondence Analysis

#### 3.4.1. Qi Deficiency and Blood Stasis Symptom Type

**Figure 3:**
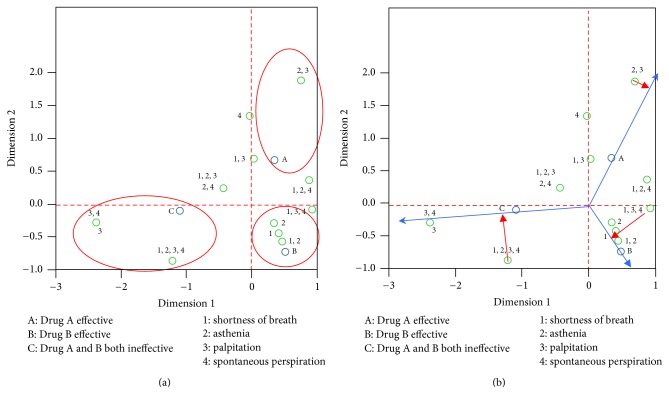
Correspondence analysis for Qi deficiency and blood stasis symptom type.

*General Observation (Figure 3(a))*. In the CA figure, draw straight lines crossing the 0 point parallel to the transverse and vertical axis, as the red dotted line in the figure, to divide the area into four areas. Homogeneous variables distributed in different areas show significant difference; distribution in the same area shows high similarity. We can see from Figure 3(a) that drug A effective (marked as A), drug B effective (marked as B), and drugs A and B ineffective (marked as C) are distributed in three different areas, manifesting significant differences between population types.

*Observation of Neighboring Areas*. Inhomogeneous variables in the same area reveal a close relationship between the type and symptom, or indicate such symptom is an indication of such efficacy. We can see from Figure 3(a) that “1, 2,” “1,” “2,” and “1, 2, 4” and drug B fall into the same area, indicating a positive relationship between shortness of breath and the effect of drug B. We can thus infer that compound danshen pills is recommended particularly for angina pectoris patients presenting shortness of breath with Qi deficiency and blood stasis syndrome; its effect is superior to that of Qishen Yiqi pills.

As shown in Figure 3(a): “1, 2, 4” symptomatic assemblage and A are in the same area, revealing a relevance between “shortness of breath, asthenia, and spontaneous perspiration” and Qishen Yiqi pills. Therefore, Qishen Yiqi pills is recommended especially for patients with angina pectoris with Qi deficiency and blood stasis syndrome and featuring clinical presentations of shortness of breath, asthenia, and spontaneous perspiration, and it works better than compound danshen pills.

Figure 3(a) shows that “3”, “3, 4”, “1, 2, 3, 4” of Qi deficiency and blood stasis symptoms and B appear in the same area, or “palpitation,” “palpitation accompanied with spontaneous perspiration,” and “palpitation, shortness of breath, asthenia, and spontaneous perspiration” are in positive relation to C, indicating that AP patients with palpitation shall select other drugs for treatment if the effect of drug A and B is undesirable.

*Vector Analysis-Preference Sequencing*. Join up the center and any point to form vector, for example, creating vectors toward A, B, and C, respectively, from the center, construct perpendicular lines from all points of symptoms toward this vector and its extension line; the closer the vertical point to the positive direction of the vector is, the more preferred this method is.

*Preference Ordering of Qishen Yiqi Pills Effective*. We can see from figure (Figure 3(b)) that A is in the upper right area and the ordering from the center to the farthest end of the positive direction of the vectors “1, 2, 4,” “4,” “13.” Thus, we can infer that Qishen Yiqi pills is more appropriate for patients with angina pectoris with symptoms such as shortness of breath, asthenia, and spontaneous perspiration.

*Preference Ordering of Compound Danshen Pills Effective*. We can see from the figure (Figure 3(b)) that B is found on the lower right part of the Figure, the ordering from the center to the farthest end of the positive direction of the vector runs in turn as 12, 1, 2, or “shortness of breath, asthenia,” “short of breath,” and “asthenia.” We can infer that compound danshen pills is more appropriate for patients with angina pectoris presenting shortness of breath and asthenia. The Figure also reveals that the effects of both drug A and B are poor for symptoms of Qi deficiency and blood stasis; the descending ordering is palpitation, palpitation with spontaneous perspiration, perspiration with asthenia, short of breath with asthenia, palpitation, and spontaneous perspiration. We come to a conclusion: other drugs instead of the compound danshen pills and Qishen Yiqi pills should be used for angina pectoris accompanied with palpitation.

#### 3.4.2. Qi Stagnation and Blood Stasis Symptom Types

**Figure 4:**
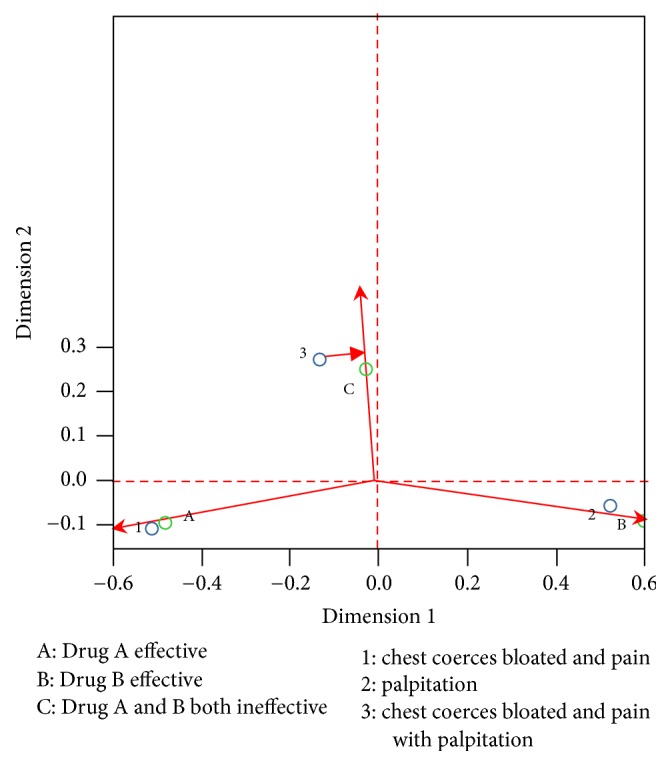
Correspondence analysis for Qi stagnation and blood stasis symptom types.

*General Observation*. We can see from Figure 4, drug A effective (marked as A), drug B effective (marked as B), and drug A and B ineffective (marked as C) are distributed in three different areas. We see that those below the central line of the transverse axis are all effective; those above it are all ineffective.

*Observing the Neighboring Area*. According to Figure 4, “1” is the most relevant to A and “2” is the most relevant to drug B. Therefore, relatively speaking, Qishen Yiqi pills works better in patients with clinical symptom of distending pain in chest; compound danshen pills works better on patients presenting palpitation.

*Vector Analysis-Preference Sequence*. We can join up a line-vector from the center to any point, for example, forming a vector from the center to A. Then construct perpendicular lines from all Qi stagnation and blood stasis toward this vector and its extension line, and vertical point 1 is the closest to the positive direction of the vector, showing better effect of A for symptom of “1.” In other words, in a relative sense, the effect of Qishen Yiqi pills is superior to that of compound danshen pills on patients with angina pectoris (Qi stagnation and blood stasis syndrome) presenting distending pain in chest.

Construct a vector from the center to B (Figure 4), draw perpendicular lines from all Qi stagnation and blood stasis symptoms toward this vector and its extension line, and vertical point 2 is the closest to the positive direction of the vector, implying better effect of B for palpitation. In other words, compound danshen pills outperforms Qishen Yiqi pills in treating patients with angina pectoris (Qi stagnation and blood stasis syndrome) manifesting palpitation.

Form a vector from the center to C (Figure 4) and construct perpendicular lines from all Qi stagnation and blood stasis symptoms toward this vector and its extension line; it can be see that vertical point 3 is the closest to the positive direction of the vector, implying poor effect of both A and B for distending pain in chest and pain with palpitation. That is, other drugs shall be considered for patients presenting distending pain in chest and pain with palpitation.

**Figure 5:**
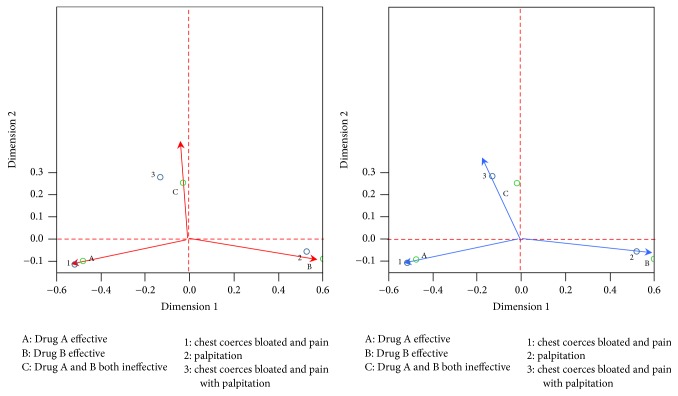
Cosine theory.

*Vector's Included Angle-Cosine Theory*. Next, we can see similarities between Qi stagnation and blood stasis symptoms or between classification outcomes from the perspective of included angle. In terms of cosine theory, the Figure reveals that when we create a vector from the center to any two points (same type), if the included angle is acute, its shows similarity between the two methods; if the angle is obtuse, it reveals significant difference, as shown in Figure 5.

In Figure 5, point 3 and point 1 form an acute angle, revealing high relevance between the two; contrarily, if the two form an obtuse angle, the relevance is low. This shows that point 1 is a major indicator of Qi stagnation and blood stasis syndrome.

#### 3.4.3. Discussion

We can come to the following conclusion from this study.

(*1) Patients with Qi Deficiency and Blood Stasis Syndrome*. Qishen Yiqi pills is advantageous over compound danshen pills in treating patients with asthenia and spontaneous perspiration; compound danshen pills is more advantageous over Qishen Yiqi pills in treating patients with shortness of breath. For patients with palpitation, the effect of both drugs is undesirable.

(*2) Patients with Qi Stagnation and Blood Stasis Syndrome*. Qishen Yiqi pills is advantageous in treating patients presenting distending pain in chest. Compound danshen pills is advantageous in treating patients with palpitation. Qishen yiqi pills is relatively more appropriate for treating AP with Qi stagnation and blood stasis syndrome over compound danshen pills.

 In conclusion, compound danshen pills and Qishen Yiqi pills have their own advantages in treating Qi deficiency and blood stasis and Qi stagnation and blood stasis syndromes of AP.

## 4. Conclusion

We have gained some insight into similar Chinese patent medicines in treating AP based on the outcomes of this study. Chinese patent medicines are modern dosages of Chinese patent medicines developed from formulas with proven clinical effect, and their composition and compatibility agree with the Chinese syndrome differentiation system [22]. Therefore, Chinese patent medicines target TCM syndromes rather than modern diseases [23]. Symptoms are basic units of TCM syndrome, or TCM syndrome consists of a series of specific clinical symptoms [24]. Meanwhile, symptoms are the easiest method for non-TCM persons to be recognized and identified, and differentiating improvement on symptoms or series of symptoms is possibly a breakthrough to differentiating clinical effect of different Chinese patent medicines. Based on the above theory and Chinese syndrome theory, this study began with improving the clinical symptoms of patients and made an analysis and comparison of the effect and respective advantages of two Chinese patient medicines Qishen Yiqi pills and compound danshen pills.

In clinical application, people shall not be absolutely positive or negative about them but shall select them for use. This will help give best play to their own therapeutic advantages, promote their merits and avoid their shortcomings, and achieve accurate use of them.

## Figures and Tables

**Table 1:** Intergroup comparison of baseline population characteristics.

Index	Group 1 (*N* = 59)	Group 2 (*N* = 60)	Statistics	*P* value
Sex				
F	30	28	0.208	0.648
M	29	32		
Age	63.25 ± 10.42	59.49 ± 11.74	1.8467	0.0673
Respiratory rate	21.71 ± 2.73	21.43 ± 2.91	0.5411	0.5895
Heart rate	71.09 ± 8.42	69.14 ± 7.94	1.3000	0.1962
Disease course	66.24 ± 10.56	66.37 ± 11.31	−0.0648	0.9485

**Table 2:** Classified outcomes.

Syndrome	Symptom type	Classified outcomes		
		Drug A effective (B effective + A ineffective but drug B effective)	Drug B effective (B effective + A ineffective but drug B effective)	Drug A, B ineffective
Qi deficiency and blood stasis syndrome	Combination 1	4	7	1
	Combination 2	3	3	1
	Combination 3	0	0	1
	Combination 4	3	0	2
	Combinations 1, 2	3	6	2
	Combinations 1, 3	5	4	3
	Combinations 1, 4	1	1	0
	Combinations 2, 3	0	3	3
	Combinations 2, 4	2	1	2
	Combinations 3, 4	0	0	1
	Combinations 1, 2, 3	2	2	1
	Combinations 1, 2, 4	1	0	0
	Combinations 1, 3, 4	2	2	0
	Combinations 1, 2, 3, 4	0	1	2

Qi stagnation and blood stasis syndrome	Combination 5	2	3	4
	Combination 6	0	1	6
	Combinations 5, 6	7	11	8

## Abbreviations

TCM: Traditional Chinese Medicine
CHD: Coronary heart disease
AP: Angina pectoris
RCT: Randomized controlled trial
CER: Comparative effectiveness research
PIO: Patient important outcome
PRO: Patient report outcome
SAQ: Seattle angina questionnaire score
LS: Likert scale
MCID: Minimal clinically important differences
CA: Correspondence analysis.

## Additional Points

*Strengths and Limitations of This Study. (1) Strengths*. (1) Strengths of the study include enriching and improving individual evaluation method of similar Chinese patent medicines and making up for the deficiency of traditional RCT; (2) promoting CUPID method and achieving precise clinical use of medication, maximizing the clinical efficacy of Chinese patient medicine, and avoiding their clinical risks through extensive application.* (2) Limitations*. There remains a need of larger scale clinical validation.

*Adverse Events*. This trial reported 3 adverse events in all, 1 diarrhea, 1 stomach pain, and 1 rash. None is relevant to the drug. No severe AE was reported.
